# Rearing in enriched environment increases parvalbumin-positive small neurons in the amygdala and decreases anxiety-like behavior of male rats

**DOI:** 10.1186/1471-2202-14-13

**Published:** 2013-01-25

**Authors:** Susumu Urakawa, Kouich Takamoto, Etsuro Hori, Natsuko Sakai, Taketoshi Ono, Hisao Nishijo

**Affiliations:** 1 Department of Judo Neurophysiotherapy, Graduate school of Medicine and Pharmaceutical Sciences, University of Toyama, Sugitani 2630, Toyama, 930-0194, Japan; 2 Department of System Emotional Science, Graduate school of Medicine and Pharmaceutical Sciences, University of Toyama, Sugitani 2630, Toyama, 930-0194, Japan

**Keywords:** Environmental enrichment, Calbindin, Calcium binding proteins, GABA, Anxiety, Amygdala

## Abstract

**Background:**

Early life experiences including physical exercise, sensory stimulation, and social interaction can modulate development of the inhibitory neuronal network and modify various behaviors. In particular, alteration of parvalbumin-expressing neurons, a gamma-aminobutyric acid (GABA)ergic neuronal subpopulation, has been suggested to be associated with psychiatric disorders. Here we investigated whether rearing in enriched environment could modify the expression of parvalbumin-positive neurons in the basolateral amygdala and anxiety-like behavior.

**Results:**

Three-week-old male rats were divided into two groups: those reared in an enriched environment (EE rats) and those reared in standard cages (SE rats). After 5 weeks of rearing, the EE rats showed decreased anxiety-like behavior in an open field than the SE rats. Under another anxiogenic situation, in a beam walking test, the EE rats more quickly traversed an elevated narrow beam. Anxiety-like behavior in the open field was significantly and negatively correlated with walking time in the beam-walking test. Immunohistochemical tests revealed that the number of parvalbumin-positive neurons significantly increased in the basolateral amygdala of the EE rats than that of the SE rats, while the number of calbindin-D28k-positive neurons did not change. These parvalbumin-positive neurons had small, rounded soma and co-expressed the glutamate decarboxylase (GAD67). Furthermore, the number of parvalbumin-positive small cells in the basolateral amygdala tended to positively correlate with emergence in the center arena of the open field and negatively correlated with walking time in the beam walking test.

**Conclusion:**

Rearing in the enriched environment augmented the number of parvalbumin-containing specific inhibitory neuron in the basolateral amygdala, but not that of calbindin-containing neuronal phenotype. Furthermore, the number of parvalbumin-positive small neurons in the basolateral amygdala was negatively correlated with walking time in the beam walking test and tended to be positively correlated with activity in the center arena in the open field test. The results suggest that rearing in the enriched environment augmented parvalbumin-positive specific neurons in the basolateral amygdala, which induced behavioral plasticity that was reflected by a decrease in anxiety-like behavior in anxiogenic situations.

## Background

Differential formation of excitatory and inhibitory synapses is critical for the functional development of the central nervous system. Inhibitory synaptic networks mediated by γ-aminobutyric acid (GABA) transmission are formed in the embryonic stages and mature as a result of neural activity and experience during the postnatal period [1,2]. A class of GABAergic interneurons is characterized by expression of calcium-binding protein, parvalbumin (PV). Most PV-positive interneurons also express the 67 kDa isoform of glutamate decarboxylase (GAD67) [3,4] and have been suggested to be involved in various higher brain functions including emotion, anxiety, and learning and memory [5-7].

The basolateral amygdala (BLA) plays a critical role in emotional responsiveness and anxiety [8,9] and approximately 10%–15% BLA neurons are GABAergic interneurons [10,11]. Of these, PV-positive neurons constitute approximately 50% of the interneuronal population and extensively colocalize with calbindin-D28k (CalB, a kind of calcium-binding protein) [12]. These PV-positive interneurons are potent inhibitors of both the perisomatic and distal dendritic domains of principal glutamatergic neurons [13]. Consequently, excitability of the principal BLA neurons is regulated by these GABAergic neurons [14-18]. Thus, PV-positive neurons are critical for the expression of anxiety-like behavior since activation of PV-positive neurons and pharmacological stimulation of the GABAergic neuronal system in BLA can decrease emotional arousal and anxiety-like behavior [7,19,20].

Rearing in an enriched environment (EE), i.e., housing conditions containing toys, tunnels, ladders, running wheel, and several animals living together, is generally believed to facilitate enhanced motor, sensory, and cognitive stimulation and also provide relatively increased social interaction than a standard environment (SE; housing conditions in normal laboratory cages). Extensive studies demonstrated significant effects of EE on brain plasticity and subsequent behavior in adulthood [21,22]. In terms of behavioral aspects, rodents reared in EE exhibit improved recovery from dysfunctions following brain lesions [23,24], enhanced learning and memory [25,26], and altered emotional behavior, such as increased exploratory and decreased anxiety-like behavior [27-29]. Taken together, these studies suggest that rearing in EE may induce functional plasticity of the PV-positive neurons in BLA. However, no previous studies have investigated the effects of EE on PV-positive neurons in BLA. Here, we investigated whether EE affected the number of PV-positive neurons in BLA and anxiety-like behavior and analyzed the relationships between these changes.

## Methods

### Animals and housing conditions

Fifteen male Wistar rats (25 days old at the start of the experiment) were used (SLC, Hamamatsu, Japan). The rats were housed in SE (2 rats in each laboratory cage, 40 × 23 × 18 cm, n = 8) or EE (7 rats in a large cage, 81 × 51 × 53 cm, n = 7). We have adopted the experimental design with the standard definition of EE, “a combination of complex inanimate and social stimulation” by van Praag *et al.*[21]. The EE was equipped with horizontal platforms and various toys such as a running wheel, tunnels, a climbing ladder, wooden blocks, a bridge, and a maze, as previously described [24,30]. The spatial arrangement of the objects was changed and some of toys were replaced with new toys twice a week in the EE cage. The rats were maintained under controlled temperature (22 ± 1°C) and 12-h light/dark cycle (lights on 7:00 AM), and food and water were available *ad libitum*. All experiments and animal housing adhered to the Guideline for Care and Use of Laboratory Animals of the Institute of Laboratory Animal Resources, National Research Council (1996). Experimental procedures were approved by the ethical review board for animal experiments at the University of Toyama (approval number; S-2009 MED-29).

### Open field test

Five weeks after rearing in each condition, activity in an open field was evaluated. The test was initiated by placing each subject in the center of an open field arena (center arena, 30 cm in diameter) of a circular open field (60 cm in diameter, surrounded by a 40 cm wall). The behavioral field was set on a black plastic sheet to obtain clear image contrast of the animals. Behavior was video recorded for 10 min using a digital video camera (Everio GZ-MG275, Victor, Kanagawa, Japan), and locomotive behavior was analyzed offline using a software program (TopScan ver 1.00, Clever Sys., Inc., VA, USA). We analyzed % distance traveled in the center arena and the time spent in the center of the arena. The % distance traveled in the center arena was defined as the following formula; 100 × [distance traveled in the center arena]/[total distance traveled]. The total distance traveled was defined as the distance traveled in the whole open field.

### Beam walking test

Two days after the open field test, beam walking ability [24,30] was evaluated. In brief, the rats were trained so as to walk on a wooden beam (25 mm wide) elevated 600 mm above the floor three times. After training, walking to traverse the beam (700 mm distance) was video recorded 3 times (Everio GZ-MG275) and walking time was calculated.

### Tissue preparation

Principle staining procedures have been previously described in detail [24]. In brief, the brains of the rats were obtained after perfusion with heparin saline solution under deep anesthesia with pentobarbital (50 mg/kg, i.p.) and fixation with 4% paraformaldehyde in 0.1 M phosphate buffer. After fixation (4% paraformaldehyde solution) and cryoprotection in 20% sucrose solution, coronal sections (40 μm thick) were cut on a freezing microtome. For stereological quantification, 5 sections each were processed for one staining each: one for Nissl staining with cresyl violet, one for PV staining, one for CalB, and two for other staining including double immunolabeling to identify the phenotype of PV-positive cells.

### Immunohistochemistry

For all staining, free-floating sections were rinsed 3 times in phosphate buffer saline (PBS) between each incubation step. These sections were quenched for 10 min in 3% H_2_O_2_/20% methanol in PBS, and incubated in a blocking solution, 3% normal horse serum in PBS-T (0.25% Triton X-100) for 30 min at room temperature. For PV and CalB immunohistochemistry, the sections were incubated overnight at 4°C with mouse anti-PV antibodies (1:10000, Sigma, St. Louis, MO, USA) or mouse anti-CalB antibodies (1:8000, Swant, Bellinzona, Switzerland) in 1% blocking solution. After rinsing, sections were incubated for 1 h at room temperature with biotinylated secondary antibodies (1:500, Vector Laboratories, Burlingame, CA, USA) and then reacted with avidin biotin peroxidase complex (ABC-Elite, Vector Laboratories). The reaction was visualized with a detection solution (0.25 mg/ml 3, 3’-diaminobenzidine, 0.03% H_2_O_2_ in PBS).

### Cell counting and morphological analysis

For cell counting, the stereological methods (optical dissector method) have been recommended [31,32]. However, poor penetration of the CalB immunostaining made it quite difficult to apply to the optical dissector method [33]. Therefore, we counted all somatic profiles contained in the optical sections 0–4 μm from the surface [33]. Images of the sections were obtained with a light microscope (BX 61, Olympus, Tokyo, Japan) equipped with a digital camera (DP 70, Olympus) or all-in-one fluorescence microscope system (BZ-9000, Keyence, Osaka, Japan). At the outset, we examined the penetration of the immunostaining for PV and CalB in 40 μm thick sections. Stacks of serial optical sections 1 μm apart were obtained with all-in-one microscope system BZ-9000 under an objective lens (× 20, NA 0.75).

We counted stained cells in the whole area of the basolateral complex of the amygdala in each section, including the lateral (dorsolateral, ventrolateral, and medial divisions), basal (magnocellular, intermediate, and parvicellular divisions), and accessory basal nuclei (magnocellular and parvicellular divisions) of the amygdala. The basolateral complex of the amygdala was divided into the 2 subareas; the lateral amygdala (LA, lateral nuclei of amygdala) and basolateral amygdala (BLA, basal and accessory basal nuclei of amygdala). Cell counting was performed for each animal in 4 anatomically matched-sections that referred to adjacent Nissl staining sections [3,34]: rostrocaudal location at (1) anterior to posterior (AP) −2.60, (2) AP −2.80, (3) AP −3.00, and (4) −3.20 mm from the bregma for PV-positive cell counting. The sections adjacent to PV-staining were used for CalB-positive cell counting. PV and CalB-positive cells were quantified separately in the SE and EE rats and expressed as mean values ± SEM (standard error of means) per section. PV-immunoreactive cells were differentially evaluated according to diameters of positive cell bodies: the cells with large cell bodies more than 25 μm rectangular diameter (length along the long axis plus those of short axis) were defined as large-positive cells, and the cells with small cell bodies less than 25 μm rectangular diameter were defined as small-positive cells. All differential counts were performed blind in randomized section. Morphological images were adjusted for brightness and contrast using Adobe Photoshop CS (v 8.0, Adobe Systems Incorporated, San Jose, CA, USA).

### Phenotype of PV-positive cells

For double-immunolabeling studies, the sections were incubated in the blocking solution, followed by overnight incubation of the sections for 2 days at 4°C with 2 primary antibodies simultaneously—rabbit anti-PV antibodies (1:1000, Calbiochem, La Jolla, CA, USA) and mouse anti-GAD 67 antibodies (1:200, Millipore, Temecula, CA, USA). The secondary antibodies were Alexa Fluor 488 goat anti-rabbit IgG (1:500, Molecular Probes, Eugene, OR, USA) and Alexa Fluor 568 goat anti-mouse IgG (1:200, Molecular Probes). The sections were mounted on MAS-coated glass slides (Matsunami, Osaka, Japan) in mounting medium with DAPI (DAPI: a nuclear counterstain for blue-fluorescence, Vector Laboratories). Immunofluorescent images were obtained using the confocal laser scanning microscope LSM 700 (Carl Zeiss, Oberkochen, Germany) and analyzed to determine whether PV-positive cells colocalize with GAD-67 using software (ZEN 2009 Light Edition, Carl Zeiss). The sections for immunofluorescence examination were located at rostrocaudal levels from AP −2.88 to AP −3.08 mm from the bregma. We confirmed that there was no non-specific labeling of cells when each primary antibody was omitted. Only double immunofluorescence-positive cells that also expressed DAPI-positive fluorescence were identified in order to avoid counting of partial and insufficient somata.

### Statistical analysis

All values are expressed as mean values ± SEM. All statistical analyses were performed using the software package SPSS (v 19, IBM, Somer, NY, USA). Significant differences between the groups in behavioral and immunohistochemical analyses were evaluated using Student’s *t*-test and two-way repeated measures ANOVA. The correlation between behavioral data and the number of small cells was analyzed using simple linear regression analysis. Differences were considered statistically significant at *p* < 0.05.

## Results

### Number of PV and CalB-positive cells in BLA

**Figure 1:**
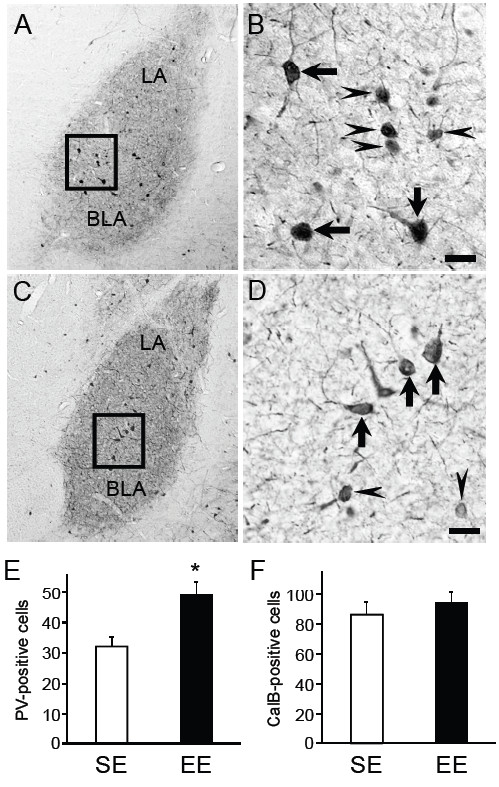
**Immunohistochemical staining for parvalbumin (PV) of EE and SE rats, and cell-counting for PV and calbindin D-28k (CalB) in the basolateral amygdala (BLA).** Photomicrographs show staining for PV of EE (**A**, **B**) and SE (**C**, **D**) rat amygdala area. High density of PV-positive neurons in BLA [the lateral (LA) and basal nuclei (BA)] was observed (A, C). B and D, higher magnification of the square area in A and C. PV-positive neurons differed in cell sizes—large immunoreactive cells (rectangular diameter of perikaryon > 25 μm, arrows) and small immunoreactive cells (rectangular diameter of perikaryon < 25 μm, arrowheads). The total number of immunoreactive cells in BLA was compared between the SE and EE rats (**E** and **F**). In the EE rats, the number of PV-positive neurons was increased (B, E), while the number of CalB-positive neurons in the EE rats was comparable to that in the SE rats (F). Data represented mean value per section. * Significant difference from SE rats, *p* < 0.05. Scale bars = 25 μm in B and D.

To determine whether rearing in EE leads to an altered organization of inhibitory circuits in BLA, we analyzed the number of cells expressing the calcium-binding proteins PV or CalB. Immunohistochemical results for PV and CalB in BLA of the SE rats were identical to those in previous studies [3,35]. BLA contained the highest density of PV-positive neurons (Figure 1A-D), whereas only a few PV-positive neurons were observed in other areas of the amygdala (e.g., medial or central amygdala). The number of PV-positive neurons in BLA was significantly higher in the rats reared in EE (EE rats) than those reared in SE (SE rats) (Student’s *t*-test, *p* < 0.05) (Figure 1E). However, no significant difference was observed between the groups in the number of CalB-positive neurons (Figure 1F).

### Cellular characterization of the increased PV-positive cells

**Figure 2:**
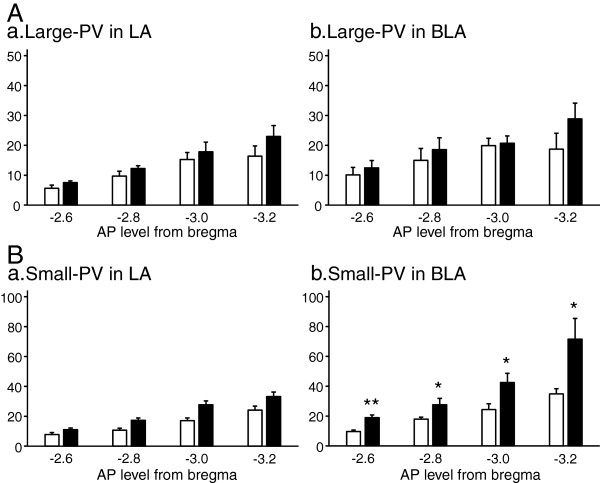
**Area- and size-dependent analyses of PV-positive neurons.** A significant difference was not found between the EE and SE rats in PV-positive large cells **(Aa, b)**. However, the number of PV-positive small cells significantly increased in EE rats both in LA (repeated measures two-way ANOVA, significant main effect of group, F (1, 13) = 16.94, *p* < 0.005, **Ba)** and BLA (repeated measures two-way ANOVA, significant main effect of group, F (1, 13) = 10.26, *p* < 0.01, **Bb)**. A significant interaction between group and AP level was found in PV-positive small cells only in BLA. *, * *, significant difference between SE and EE (Bonferroni tests, *p* < 0.05, *p* < 0.001, respectively) **(Bb)**. The white bars indicate the number of PV-positive cells for SE rats, and black bars indicate those for EE rats.

PV-positive cells consist of various sub-types with different somata sizes (see Methods). We separately counted the neurons according to cell sizes, large or small positive cells. Statistical comparisons indicated that there was no significant difference in the number of the large PV-positive cells (rectangular diameter > 25 μm) between the groups in LA; there was no significant main effect of group (repeated measures two-way ANOVA, F (1, 13) = 2.09, *p* > 0.05) and no significant interaction between group and AP level (repeated measures two-way ANOVA, F (3, 39) = 0.61, *p* > 0.05) (Figure 2Aa). Furthermore, there was no significant difference in the number of the large PV-positive cells between the groups in BLA; there was no significant main effect of group (two-way repeated measures ANOVA, F (1, 13) = 1.66, *p* > 0.05) and no significant interaction between group and AP level (two-way repeated measures ANOVA, F (3, 39) = 1.45, *p* > 0.05) (Figure 2Ab).

On the other hand, statistical analyses showed that EE significantly increased the number of the small PV-positive cells (rectangular diameter < 25 μm) in LA; there was a significant main effect of group (repeated measures two-way ANOVA, F (1, 13) = 16.94, *p* < 0.005), but no significant interaction between group and AP level (F (3, 39) = 1.78, *p* > 0.05) (Figure 2Ba). Furthermore, EE also significantly increased the number of the small PV-positive cells in BLA; there were a significant main effect of group (repeated measures two-way ANOVA, F (1, 13) = 10.26, *p* < 0.01), and a significant interaction between group and AP level (repeated measures two-way ANOVA, F (3, 39) = 4.18, *p* < 0.05) (Figure 2Bb). Post-hoc tests indicated that the number of the small PV-positive cells was significantly larger in EE than SE in the all AP levels (Bonferroni tests, *p* < 0.05 or *p* < 0.001.

**Figure 3:**
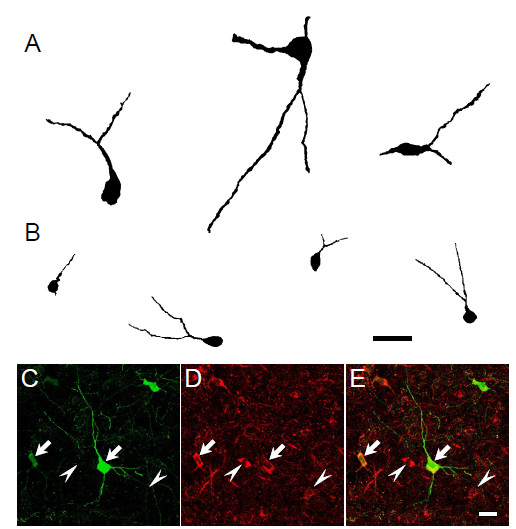
**Morphological and immunohistochemical features of PV-positive neurons in BLA.** Drawing of PV-positive neurons with large perikaryon (**A**) and small perikaryon (**B**). Almost all PV-positive neurons (**C**) co-expressed GAD 67 (**D**) (**E**, merged image) in both types of neurons with large and small perikaryon. Arrows indicate both (PV and GAD67) immunoreactive cells and arrowheads indicate single immunoreactive cells of GAD67 alone. Scale bars = 25 μm in B and E.

Based on the characteristics of the somata and dendritic tree, neurons expressing calcium-binding proteins have been classified into 4 major morphological types [36,37]. In PV-positive neurons of BLA, Kemppainen and Pitkänen have reported 3 predominant morphological types of neurons (types 1–3) [3]. According to our classification based on cell size, large positive cells exhibited 2 morphological features (Figure 3A): one type was medium-to-large-sized multipolar neurons with a few longer, aspiny dendrites of varying thickness, and corresponded to type 2 cells; the other type had fusiform somata with elongated dendrites and corresponded to type 3 cells. PV-positive small cells had small, roundish somata or multipolar stellate perikarya with a few shorter dendrites of approximately equal thickness, which corresponded to type 1 cells (Figure 3B). The morphological features of the PV-positive neurons according to our classification based on cell size were consistent with those according to the previously reported classification based on cell type (type 1–3) [3].

Almost all PV-positive neurons in BLA are GABAergic (including GAD-positive) [3,4]. However, in postmortem specimens of subjects with schizophrenia, only 55% PV mRNA-positive neurons showed detectable levels of GAD67 mRNA in the prefrontal cortex [38]. Accordingly, EE and/or SE may also affect co-expressing phenotype for GAD67 in PV-positive cells. Therefore, to determine whether the increased population of PV-positive cells co-expressed GAD67, 991 PV-positive cells of 8 SE rats and 1042 PV-positive cells of 7 EE rats were analyzed for colocalization with GAD67 in the BLA. Among the PV-positive cells (Figure 3C), 99.3 ± 0.2% in SE rats and 99.5 ± 0.3% in EE rats, were immunoreactive for co-expression of GAD67 (Figure 3D and E). No significant difference was observed in colocalization between the SE and EE rats (data not shown). The pattern of colocalization did not differ in the counted areas (lateral, basal, and accessory basal nuclei) or in cell-type classification (large or small positive cells) (data not shown). Thus, although the number of PV-positive small cells was increased in the EE rats, the results indicate that the morphological and immunohistochemical phenotypes of the PV-positive cells did not show significant difference between the SE and EE rats.

### Behavioral alteration between the rearing groups

**Figure 4:**
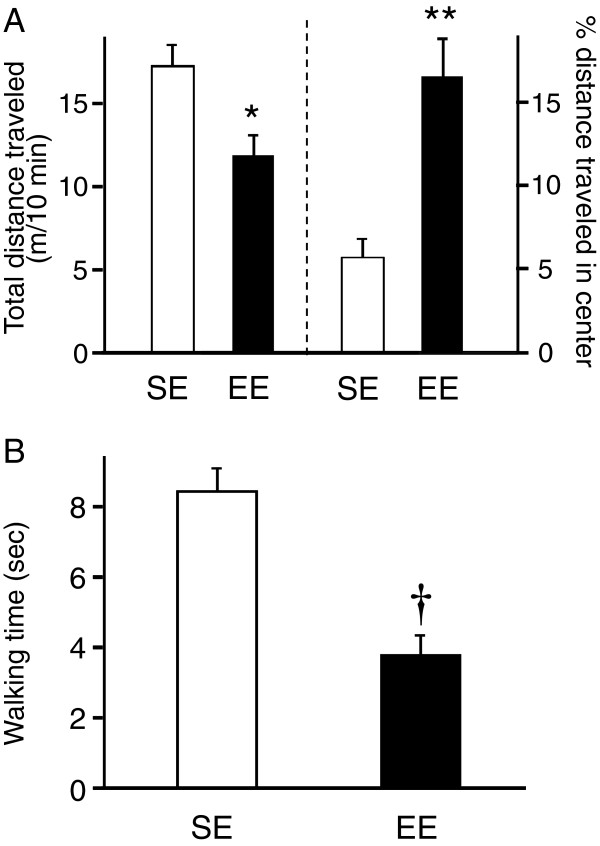
**Behavioral assessment in the open field (A) and beam walking (B) tests.** In the open field test, the EE rats showed lower locomotive activity in the total distance traveled (A, left) and lowered anxiety reflected in the % distance traveled in the center arena (A, right). In the beam walking test, the EE rats quickly traversed the elevated narrow beam (B). *, **, †, significant difference from SE rats, *p* < 0.01, 0.005, 0.001, respectively.

If the increased PV-positive cells were integrated into the neuronal circuits of BLA, some behavioral alterations would be induced because BLA plays a critical role in the expression of anxiety-like behaviors under stressful situations [8,9]. To validate this hypothesis, we compared behavioral phenotypes between the EE rats, which increased the number of PV-positive cells in BLA, and SE rats. In the open field test, the total distance traveled was significantly shorter in the EE rats than the SE rats (Student’s *t*-test, *p* < 0.01; Figure 4A, left panel). In addition, separate analyses indicated that the distance traveled in the peripheral arena was also significantly shorter in the EE rats than the SE rats (Student’s *t*-test, *p* < 0.005), whereas the distance traveled in the center arena was significantly greater in the EE rats than the SE rats (Student’s *t*-test, *p* < 0.05). Although the EE rats exhibited decreased locomotion than the SE rats, this trend was evident only in the peripheral arena. Thus, the % distance traveled in the center arena was significantly greater in the EE rats than the SE rats (Student’s *t*-test, *p* < 0.005; Figure 4A, right panel). Furthermore, the time spent in the center arena significantly increased in the EE rats than the SE rats (Student’s *t*-test, *p* < 0.01).

**Figure 5:**
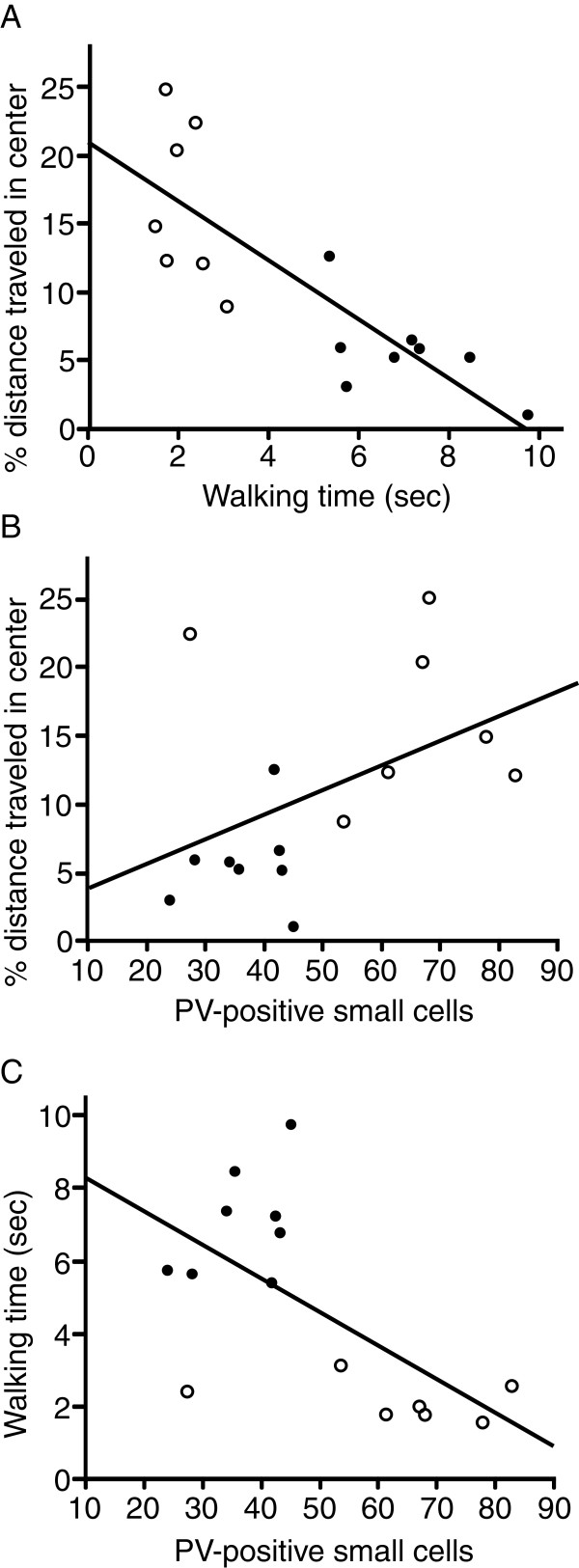
**Relationship between behavioral alterations (open field and beam walking tests) and PV-positive cells in BLA.** Simple regression analysis indicated that walking time in the beam walking test was significantly and negatively correlated with the % distance traveled in the center arena in the open field test (**A**, *p* < 0.001). Simple regression analysis also revealed that the number of PV-positive small cells tended to be positively correlated with the % distance traveled in the center arena in the open field test (**B**, *p* = 0.078) and significantly and negatively correlated with walking time in the beam walking test (**C**, *p* < 0.05). Open circles, EE; closed circles, SE.

In a previous report, EE rats showed a good performance in a beam walking task [30]. In the present study, the EE rats also traversed the elevated narrow beam faster (Student’s *t*-test, *p* < 0.001; Figure 4B). Because the beam was narrow and elevated, the beam walking test was also anxiogenic. Therefore, performance in both anxiogenic tasks (open field and beam walking tests) should be correlated. Simple regression analysis revealed that walking time was significantly and negatively correlated with the % distance traveled in the center arena (F (1, 14) = 25.2, *p* < 0.001, *R* = 0.81; Figure 5A) and tended to be positively correlated with the total distance traveled (F (1, 14) = 4.1, *p* = 0.064, *R* = 0.49).

### Relationships between behavioral alteration and increased number of PV-positive cells

The observed alterations of PV-positive cells in BLA could affect behavior in the anxiogenic (open field and beam walking) tests. Figure 5B and C shows the relationships between performance in these behavioral tests and the number of PV-positive small cells in BLA. Simple regression analyses revealed that the number of PV-positive small cells tended to be positively correlated with the % distance traveled in the center arena (F (1, 14) = 3.7, *p* = 0.078, *R* = 0.47; Figure 5B), and negatively correlated with the peripheral distance traveled (F (1, 14) = 3.2, *p* = 0.097, *R* = 0.44). Furthermore, a simple regression analysis also revealed that the number of PV-positive small cells tended to be positively correlated with the time spent in the center arena (F (1, 14) = 4.0, *p* = 0.067, *R* = 0.49). On the other hand, the number of PV-positive small cells was not significantly correlated with the total distance traveled (F (1, 14) = 2.1, *p* > 0.1, *R* = 0.38) nor with the center distance traveled (F (1, 14) = 2.9, *p* > 0.1, *R* = 0.43). Notably, in the beam walking test, the number of PV-positive small cells was significantly and negatively correlated with walking time (F (1, 14) = 8.3, *p* < 0.05, *R* = 0.62; Figure 5C).

## Discussion

Our results indicate that EE significantly affects anxiety-like behavior and PV-positive neurons in BLA. First, EE increased the number of PV-containing neuronal phenotype in BLA, but not that of CalB-containing neuronal phenotype. This increase in the number of PV-positive neurons was evident only in the morphologically small neurons co-expressing GAD67. Finally, the number of small PV-positive neurons in BLA was negatively correlated with walking time in the beam walking test and tended to be positively correlated with activity in the center arena in the open field test.

### Effects of enriched environment on PV-positive neurons in BLA

In the present study, the number of PV-positive neurons in BLA was influenced by the environmental conditions during adolescence, while that of CalB-positive neurons was unchanged. A neuroanatomical study in BLA reported that PV-immunoreactivity reached maturity (in terms of the number of immunoreactive neurons) on postnatal day 30 at the same level of postnatal day 90 [39]. In the present study, we divided the animals into 2 groups (SE and EE) from postnatal day 25, and the animals were kept under same environment (SE and EE) for five weeks. These findings suggest that the number of PV-positive cells was stable in SE group, while PV-positive cells in EE increased during environmental intervention for five weeks. Furthermore, the postnatal maturation of PV-positive neurons was later than that of CalB-positive neurons [39], and some studies have reported partial phenotypic shift from PV-negative immunoreactive neurons to PV-positive neurons during postnatal development [40,41]. These results further suggest that the increase in the number of PV-positive neurons as a result of EE might be partly attributable to the phenotypic shift into PV-positive expression. Since the number of CalB-positive neurons was unchanged in the present study, two possibilities of the phenotypic shift could be raised; a shift from PV-negative and CalB-negative neurons to PV-positive and CalB-negative neurons or a shift from PV-negative and CalB-positive neurons to PV-positive and CalB-positive neurons.

Exposure to EE and physical activity (voluntary running) induces hippocampal neural progenitor proliferation and neurogenesis [21,22]. Furthermore, EE has positive effects on neurogenesis in the subventricular zone in rodent damaged brains [24,42]. These previous findings suggest that neurogenesis of PV-positive neurons might also contribute to PV-positive augmentation in the present study. However, it is unlikely since BLA is one of the non-neurogenic areas [43,44]. Neurogenesis throughout adulthood in the mammals has been clearly demonstrated at the two restricted brain areas, the subventricular zone of lateral ventricles and the subgranular zone of the dentate gyrus in the hippocampus. Although neural stem cells or progenitors can be isolated from many areas of adult nervous system, adult neurogenesis (differentiation and integration of newly born neuron) has only been consistently found in these two areas by specific neurogenic niche, but not in BLA [43,44].

A previous study reported that EE did not increase the number of PV-positive neurons [45]. The discrepancy may be partially attributable to the differences from our study: 1) the rearing conditions of the SE rats in that study–6 rats, including both males and females were housed together [45], while 2 male rats were housed in a single cage in the present study, 2) PV-positive neurons were counted in the hippocampus in that study [45]. Our findings of an increase in PV-positive neurons are consistent with the effects of physical exercise (running) on hippocampal PV-positive neurons during adolescence [46,47]. Enhanced motor and sensory experiences in the present study, in particular the use of a running wheel, may play a substantial role in modulation of PV-positive neurons in BLA.

### Effects of enriched environment on anxiety-like behavior

In the present study, the % distance traveled in the center arena in the open field test was significantly greater in the EE rats (Figure 4A). However, the total distance traveled was decreased in the EE rats (Figure 4A). This decrease in the total distance traveled was attributable to a decrease in the distance traveled only in the peripheral arena, whereas the distance traveled in the center arena was increased in the EE rats (see Results). These results suggest that rearing in EE decreased the expression of anxiety-like behavior in the rats.

In the beam walking test, walking time was shorter in the EE rats (Figure 4B). In this test, the animals were tested under the anxiogenic environment, i.e., leaving them alone on the elevated narrow beam. In humans, similar situations, such as unprotected, open, and elevated areas, can evoke panic and vestibular symptoms (vertigo and dizziness) [48-51]. These findings indicate the pathological link between anxiety and vestibular/balancing deficits [52]. These deficits are conceptualized as stress-evoked sensorimotor disintegration [53,54]. Taken together, shorter walking time in the EE rats might be attributable to changes in emotional states. In other words, decreased anxiety-like behavior in the EE rats ameliorated stress-evoked sensorimotor disintegration. Furthermore, walking time was significantly and negatively correlated with the % distance traveled in the center arena, thereby suggesting that anxiety-like behavior is negatively correlated with performance in the beam walking test (Figure 5A). These findings strongly suggest that shorter walking time in the EE rats was attributable to decreased anxiety-like behavior as a result of being reared in EE.

Other previous data also supports this hypothesis. First, the SE rats could walk faster within a few days and achieved the same level as in the EE rats, suggesting that shorter walking time in the EE rats was not attributable solely to plastic changes in only the motor system. Second, in the training session, when the EE rats were placed on a beam just in front of a goal cage (i.e., in a situation in which fine motor coordination was not required for running on the beam), they could return more quickly into the cage [30], suggesting that the EE rats could recognize the novel situation and adapt more quickly than the SE rats under the same anxiogenic conditions. These findings suggest that the plastic changes occur primarily in the brain regions involved in the expression of anxiety.

### Functional roles of PV-neurons in anxiety-like behavior

The above behavioral changes were correlated with the number of PV-positive neurons in BLA. The number of PV-positive small cells in BLA was negatively correlated with walking time in the beam walking test (Figure 5C) and tended to be positively correlated with the % distance traveled in the center arena in the open field test (Figure 5B). Because BLA is one of the brain regions involved in the expression of anxiety, the data suggest that the increased number of PV-positive neurons decreased anxiety levels in the EE rats. These results are consistent with those of a previous study [55] in which one inbred rat line that exhibited lesser anxiety-like behavior in an open field and elevated plus-maze had more PV-positive neurons in BLA than the other rat line that exhibited anxiety-like behavior. On the other hand, mice lacking ß3 subunits of GABA-A receptors exhibited pronounced neurological and balance deficits as well as hyperemotionality [56]. Furthermore, antidepressants such as benzodiazepines ameliorate both anxiety and vestibular/balancing deficits [50]. These results strongly support the hypothesis that the GABAergic subpopulation co-expressing PV in BLA is specifically responsible, at least in part, for suppressing anxiety-like behavior in the open field and beam walking tests.

### Significance of rearing in enriched environment

Our results indicate that the GABAergic system (PV-positive neurons) in BLA is augmented by EE. Deficits in PV-positive interneurons have been suggested to be associated not only with anxiety disorders (present results) but also with other psychiatric disorders. Interneurons expressing PV exhibit the fast-spiking phenotype and have been suggested to be involved in gamma oscillations [5,6]. Disruption of gamma oscillation has been suggested to underlie psychiatric disorders such as schizophrenia [57] and autism [58] and may be attributable to deficits in GABAergic PV-positive interneurons [57,59,60]. Furthermore, adverse prenatal or early life events such as exposure to ethanol and maternal separation alter emotional responsiveness and the number of PV-positive interneurons in BLA, medial septum, medial prefrontal cortex, and hippocampus [61-64]. In addition, PV-positive interneurons in BLA express excitatory 5-HT2A receptors [65], and 5-HT inhibits glutamatergic projection neurons involved in anxiety behavior through this subtype of the interneurons [66]. Therefore, our results suggest that EE, even in adolescence, might be beneficial to disorders that are characterized by deficiencies in PV-positive neuronal networks.

## Conclusions

The present results demonstrated that rearing in EE augmented a specific subpopulation of GABAergic neurons co-expressing PV in BLA. The EE rats showed lowered anxiety-like behavior in the open field test and quickly traversed the beam in the beam walking test. Significant correlation between increased numbers of PV-positive neurons in BLA and changes in anxiety-like behavior suggest that plastic changes in PV-positive neurons induced alteration of these anxiety-like behaviors under anxiogenic situations. Furthermore, rearing conditions during adolescence critically affect the inhibitory neuronal networks of the PV-positive subpopulation in BLA leading to behavioral plasticity in emotional responsiveness. These results suggest that EE might be beneficial for certain psychiatric disorders in which GABAergic dysfunction is suspected.

## Abbreviations

GABA: γ-Aminobutyric Acid; PV: Parvalbumin; CalB: Clbindin-D28k; BLA: Basolateral Amygdala; LA: Lateral Amygdala; GAD67: 67 kDa isoform of glutamate decarboxylase.

## Competing interests

There are no competing interests.

## Authors’ contributions

SU and HN conceived and designed the experiments. SU, KT, and NS performed the experiments. SU and HN analyzed the data. EH and NS contributed reagents/materials/analysis tools. SU, TO, and HN wrote the paper. All authors read and approved the final manuscript.
